# Testing the implementation of an electronic process-of-care checklist for use during morning medical rounds in a tertiary intensive care unit: a prospective before–after study

**DOI:** 10.1186/s13613-015-0060-1

**Published:** 2015-08-04

**Authors:** Karena M Conroy, Doug Elliott, Anthony R Burrell

**Affiliations:** NSW Intensive Care Co-ordination and Monitoring Unit, Agency for Clinical Innovation, Chatswood, Australia; Faculty of Health, University of Technology Sydney, PO Box 123, Broadway, NSW 2007 Australia

**Keywords:** Critical care, Checklists, Health-care quality improvement, Patient safety, Process quality

## Abstract

**Background:**

To improve the delivery of important care processes in the ICU, morning ward round checklists have been implemented in a number of intensive care units (ICUs) internationally. Good quality evidence supporting their use as clinical support tools is lacking. With increased use of technology in clinical settings, integration of such tools into current work practices can be a challenge and requires evaluation. Having completed preliminary work revealing variations in practice and evidence supporting the construct validity of a process-of-care checklist, the need to develop, test and further validate an e(lectronic)-checklist in an ICU was identified.

**Methods:**

A prospective, before–after study was conducted in a 19-bed general ICU within a tertiary hospital. Data collection occurred during baseline and intervention periods for 6 weeks each, with education and training conducted over a 4-week period prior to intervention. The e-checklist was used at baseline by ICU research nurses conducting post-ward round audits. During intervention, senior medical staff completed the e-checklist after patient assessments during the morning ward rounds, and research staff conducted post-ward round audits for validity testing (via concordance measurement). To examine changes in compliance over time, checklist-level data were analysed using generalised estimating equations that factored in confounding variables, and statistical process control charts were used to evaluate unit-level data. Established measures of concordance were used to evaluate e-checklist validity.

**Results:**

Compliance with each care component improved significantly over time; the largest improvement was for pain management (42% increase; adjusted odds ratio = 23, p < 0.001), followed by glucose management (22% increase, p < 0.001) and head-of-bed elevation (19% increase, p < 0.001), both with odds ratios greater than 10. Most detected omissions were corrected by the following day. Control charts illustrated reduced variability in care compliance over time. There was good concordance between physician and auditor e-checklist responses; seven out of nine cares had kappa values above 0.8.

**Conclusion:**

Improvements in the delivery of essential daily care processes were evidenced after the introduction of an e-checklist to the morning ward rounds in an ICU. High levels of agreement between physician and independent audit responses lend support to the validity of the e-checklist.

**Electronic supplementary material:**

The online version of this article (doi:10.1186/s13613-015-0060-1) contains supplementary material, which is available to authorized users.

## Background

The need for improvement in the delivery of important care processes in ICUs has been demonstrated internationally [1–3], highlighting a gap between evidence and practice on a wide scale [4]. Improvement initiatives designed to address this [5] can lead to improved health outcomes for patients [6]. Evidence of omissions in care highlights a need for clinical support tools to enhance work practices and the delivery of routine care [7, 8]. The use of “best practice” checklists during patient care rounds in the ICU has been identified in a recent systematic review as one of several factors that could improve the quality of service delivery [9]. While there is growing support for the use of process-of-care checklists in ICUs [9, 10], their actual contributions to improvements in patient care remain unclear due to methodological limitations of published studies [11, 12]. Integration of clinical support tools such as checklists into developing technologies has also been highlighted as important for current and evolving future practice [10, 12].

To address previous study limitations and add to the evidence base, initial development and validation of a process-of-care checklist was conducted to ensure that checklist use corresponded with delivery of care [13], and content was relevant, with clear, concise, and instructive statements for use by intensive care physicians during morning ward rounds [14]. These studies provided initial supporting evidence of the checklist’s construct validity.

After this preliminary work, development, testing and further validation of an e(lectronic)-checklist in an ICU was required. This included measurement of care delivered before and after checklist implementation to determine whether checklist use improved actual delivery of care. Inclusion of an audit function in the e-checklist would also enable evaluation of whether the checklist was being used as intended, contributing important information related to response processes—a key source of evidence required to establish construct validity [15].

Importantly, prospectively evaluating the impact of an e-checklist on patient care measures over time would address identified limitations and gaps in the current literature: lack of compliance measurement with checklists and related care processes, lack of baseline data for comparisons, retrospective study designs, small or unknown sample sizes, and a paucity of validity testing [11].

The overall study aim was to test the implementation of an e-checklist designed to facilitate patient safety and quality of care during medical ward rounds in an ICU. The specific study questions were:

1. Is there a significant difference in compliance with applicable care processes following implementation of an e-checklist?
2. What is the level of concordance between checklist item completion by physicians on the ICU ward rounds and actual delivery of care?

## Methods

### Design

A prospective, mixed-methods design with a nested before–after intervention component was used to address the research questions. This approach combined quality improvement (QI) principles, [16] methods of knowledge translation [17], and point-of-care technology [18] to implement and evaluate the electronic process-of-care checklist. The focus of this paper is on compliance with care processes and concordance between respondents to the checklist. Process data that directly informed the quality of patient care were collected daily to evaluate the utility of the checklist as a tool for use during the morning ward rounds in an ICU.

### Setting

The study site was a combined 19-bed general ICU and high dependency unit (HDU) within a tertiary hospital located in Metropolitan NSW, Australia. The unit operated under a closed medical model with patients admitted under the care of intensive care specialist physicians. A 1:1 nurse-to-patient ratio was the model of care used (1:2 for high dependency patients). At the time of the study, the ICU was funded for 13 ICU beds and 5 high dependency beds, though in practice patient mix was flexible. Annual throughput was 1,318 patient admissions, with 931 patient episodes having a length of stay greater than 24 h.

The unit was separated into two physical pods, both with central nursing stations. During morning ward rounds, the medical staff were divided into two groups, each commencing in a different pod. During the study period, each ward round team usually consisted of one consultant physician and/or senior registrar, a registrar and one or two junior medical officers.

### Participants

Each participant involved in completion of the e-checklist was a senior medical officer (intensive care physician, senior registrar or registrar). Recipients of the checklist were all applicable adult ICU patients (aged 16 years and over) admitted to the ICU during the study periods. A checklist was completed for each patient once per day during morning rounds; patients not present at the time of morning rounds (e.g. for procedure) were excluded for that day.

### Recruitment frame and sample size calculations

The primary outcome of interest was compliance with the process-of-care checklist. To examine the significance of change in rates over time, a priori power calculations were computed for overall compliance with checklist statements. A previous multi-site study [19] found compliance rates prior to intervention of 34.2% and post-intervention 56.7% in a total sample of 7,688, equating to an odds ratio (OR) of 2.52 (95% CI 2.30–2.76). Using this OR, sample size calculations for comparing two proportions were conducted (Power Analysis & Sample Size, version 12.0.2; NCSS Statistical Software, LLC. Kaysville, Utah, USA). With checklist item compliance as the outcome variable and time (baseline or follow-up) as the predictor variable, 206 participants were required to detect an odds ratio of 2.5 with a power of 0.90 and alpha set at 0.05. Based on the throughput of the study ICU for patients with a length of stay >24 h, it was estimated that 6 weeks each of baseline and post-intervention measurement would result in 214 patients, sufficient to detect clinically significant differences in compliance with process-of-care components.

### Intervention

The e-checklist was designed as a practice delivery tool with a series of prompts (via a handheld device) during the clinical round. The handheld device was a Palm TX™ personal digital assistant (PDA), the most suitable portable device for software programming and utility by clinicians at the bedside in 2009 [20]. All data collected via the PDA were sent wirelessly to a dedicated server for storage and processing. Both the PDA (acting as a thin client) and server applications were purpose built using Java technology (Sun Microsystems—now owned by Oracle, California, USA).

The e-checklist contained nine core ‘process-of-care’ statements (see Table 1), for the medical team to explore for each individual patient (i.e. the checklist was not designed to replace clinical decision-making). Content development and early validation of the checklist statements have been described previously [11, 13, 14]. The e-checklist was used during medical morning ward rounds to document either the delivery or clinical reasons for non-delivery of cares (response options outlined in Table 1; note that all items had a ‘not applicable’ (NA) option, except for the ‘ventilated’ item). Use of an ‘auditor version’ of the e-checklist enabled independent audit of whether identified care processes were implemented during the round. For the NA option, the independent auditor reviewed the patient documentation and/or confirmed with the patient’s nurse who was involved in patient care discussions during the medical round that the care was not clinically appropriate at the time.

**Table 1:** Process-of-care statements in the e-checklist

Label	Statement	Response options^a^	Inclusion criteria
Ventilated^b^	Is the patient invasively ventilated?	Yes/No	Patients with endotracheal or tracheostomy tubes only
HOB	Patient is positioned with the head of the bed raised >30 degrees	Yes/No/NA (not ventilated or unit policy e.g. patient haemodynamically unstable or needing large doses of noradrenaline, or has unstable spinal or pelvic injuries)	Invasively ventilated patients only
Wean	Patient’s readiness to be weaned from mechanical ventilation has been assessed	Yes/No/NA (not ventilated)	Invasively ventilated patients only
Sedation	Sedation target set, sedation level assessed and managed	Yes/No/NA (not ventilated or has not required sedation in the past 24 h)	Patients who have an artificial airway and require sedation for facilitation of ventilation
Analgesia	Pain has been assessed, a management plan set and progress reviewed	Yes/No/NA (pain assessment cannot be determined due to patient’s condition)	All patients—includes recognition that a patient has no pain or it cannot be determined, e.g. patient is unresponsive
DVT_proph	Mechanical and/or drug DVT prophylaxis is being administered or applied	Yes/No/NA (clinical contraindication to both forms of prophylaxis)	All patients
SUP	Stress ulcer prophylaxis is being administered	Yes/No/NA (unit policy, e.g. patient is stable and tolerating enteral feeds)/clinical contraindication	All patients mechanically ventilated (invasive or non-invasive) for >48 h
Feeding	Nutrition goals have been formally assessed and progress reviewed	Yes/No/NA (goals do not need to be assessed or reviewed, e.g. fasting for surgery)	All patients
Glucose	Blood sugar levels (BSL) have been assessed, limits have been set and are being managed to achieve those limits	Yes/No/NA (if clinically appropriate not to monitor frequently)	All patients
Meds	All medications have been checked and reviewed	Yes/No/NA (for auditors only, i.e. unable to determine)	All patients

^a^Clicking on ‘No’ prompted a ‘Reason for no’ pop-up box which included ‘omission—now corrected’, ‘omission—not yet corrected’ and the other ‘not applicable’ (NA) and ‘clinical contraindication’ responses as outlined in the table.

^b^This question was used to filter out questions that were not applicable to patients who were not invasively ventilated, i.e. if ‘no’ was selected, head-of-bed elevation, readiness to wean and sedation were auto-filled to ‘NA’.

### Study procedure

The key features of the study procedure are described below (see Additional file 1 for a more complete description).

#### Pre-baseline

Engagement of ICU clinical staff was critical to successful implementation of the e-checklist. Two senior intensivists agreed to be clinical champions and research nurses were engaged for collection of audit data. Activities included audit data collection training, software development and testing, and observations of morning ward rounds (see Additional file 2).

#### Baseline

For a period of 6 weeks (April–June 2009), an audit of morning medical ward rounds identified current practices, with data collected by research nurses (clinically trained in intensive care and with no direct patient care responsibilities) using the e-checklist audit tool 7 days a week, to ensure the audit encompassed all medical rotations. Each audit was conducted independently after completion of the ward rounds; patient medical records were checked and bedside nurses were consulted as required for accuracy and to minimise potential confounders.

#### Pre-intervention

A 4-week period between baseline data collection and intervention was used to prepare ICU and research staff for the intervention. This included providing general information to all staff participant education and training, preparing detailed instruction booklets for all participants, refining the e-checklist software and further testing of the e-checklist.

Results of the baseline audit and other supporting data [4] were shared during a medical staff meeting to facilitate project engagement. All staff were informed that the project was testing the utility of the e-checklist in delivering care, and was not an audit of individual practice. Detailed one-on-one training with all medical participants enabled tailoring for varying levels of knowledge and experience with PDAs and wireless technology.

All study participants were issued with detailed instruction booklets specific to each of their roles, highlighting checklist statements, response options, data definitions and detailed instruction (including screen shots) on e-checklist use. The data definitions were informed by previous validity work [13, 14] and consultations with ICU research staff and local intensive care physicians.

#### Intervention

For a period of 6 weeks (July–August 2009), senior medical staff members completed the e-checklist during the morning ward round for all patients in the ICU, at the end of each patient assessment as a ‘challenge-and-answer’ tool. During the post-round audits, research nurses independently collected process data 4 days a week using the e-checklist, to verify physician responses (i.e. validity testing).

### Data management and analysis

All e-checklist data were transmitted to a specifically designed networked database via a secure dedicated server where it was accessed for data management and analysis. Patient demographic and clinical data were obtained from a separate ICU database, with data linkage via unique patient identifiers (e.g. medical record number, date of birth, dates of ICU admission and discharge). Checklist-level data were then combined with patient-level data, de-identified and transferred into an SPSS database (version 17; IBM SPSS Statistics, Chicago, IL, USA) for analyses. Missing data were excluded from analyses.

Patient-level data were described by means and standard deviations for normally distributed data, medians and inter-quartile ranges for non-normally distributed data, and percentages for categorical data. Sample characteristics for baseline and intervention patient groups were compared using: (1) independent *t* test for normally distributed interval data; (2) Mann–Whitney U test for non-normally distributed data; and (3) Pearson’s Chi square for categorical data.

For each process-of-care, generalised estimating equations (GEE) analyses were conducted to examine change in compliance rates over time (adjusted for potential confounding variables) (see Additional file 3). All ‘not applicable’ checklist responses (e.g. clinical contraindication) were excluded from analyses. Statistical process control (SPC) charts were produced to evaluate compliance data at the unit level over time, highlighting stable and predictable (common cause variation) or unstable and unpredictable (special cause variation) processes [21]. Special causes were anomalies flagged using established SPC chart rule violations (see Additional file 3) [22]. The numerator for daily compliance was the sum of all ‘Yes—care delivered’ responses; the denominator was the sum of all applicable responses.

To test validity of the e-checklist, established measures of concordance (agreement between two observation sets) were used to compare physician and audit responses. Analyses were conducted on data where audits had been completed and patients who were not applicable for a care (during ward round or audit) were excluded. Concordance was assessed by: proportion of observed agreement; Byrt’s [23] kappa (measures the relationship between two respondent groups, corrected for bias); prevalence (when one response is more probable than another) and bias (when marginal distributions for the raters are unequal) indices [23]; and proportions of positive and negative agreement [24] (see Additional file 3).

The Human Research Ethics Committee approval was obtained from the health service and university. Staff participants provided informed consent prior to study involvement. The need for individual patient consent was waived by both committees, as the study was considered a quality assurance project.

## Results

### Patient sample

During the 12-week study period (6 weeks each of pre- and post-intervention data collection), 293 patients were admitted to the ICU—141 at baseline and 152 at intervention. Patient characteristics across the before–after study periods were comparable (see Table 2).

**Table 2:** Patient demographic and clinical characteristics

Variable	Baseline (n = 141)	Intervention (n = 152)	P-value
Gender (male)	57%	55%	0.73
Age^a^	57 (21)	57 (18)	0.79
APACHE III score	56 (37–76)	57 (37–79)	0.67
ICU LOS (days)	3 (2–6)	2 (1–6)	0.08
Hospital LOS (days)	10 (5–75)	11 (6–23)	0.90
Checklist days (per patient)	2 (1–5)	3 (2–5)	0.53
Mechanical ventilation hours	72 (14–165)	77 (20–194)	0.49
% Mechanically ventilated	50%	48%	0.82
Crude^b^ ICU mortality	7.8%	7.9%	1.00
Crude^b^ hospital mortality	11.4%	9.2%	0.57
ICU re-admissions	4.3%	6%	0.60
ICU:HDU admissions (%)	63:37	63:37	1.00
Emergency:elective (%)	77:23	80:20	0.48
Non-operative:post-operative	64:36	72:28	0.13
Diagnosis on admission (%)			
Respiratory	27.7	37.5	0.08
Gastrointestinal	13.5	11.2	0.60
Neurological	13.5	9.9	0.37
Sepsis	7.8	13.8	0.13
Cardiovascular	8.5	9.2	1.00
Metabolic	9.9	7.2	0.53
Trauma	10.6	3.3	0.19
Genitourinary	3.5	5.9	0.42
Gynaecological	2.8	2.0	0.71
Musculoskeletal/skin	1.4	0	0.23
Haematological	0.7	0	0.48

All patient demographic data were obtained from the ICU database.

^a^Descriptive data for age are mean and standard deviation (normal distribution); other (no normal distribution) interval data use median and inter-quartile range.

^b^Percentage of ICU admissions of patients who died in ICU or in hospital.

### Checklist compliance

From these 293 patients, 1,212 valid checklist records were generated: 635 during baseline (across 43 consecutive audit days) and 577 during intervention (generated by physicians across 41 consecutive days with 333 corresponding audit responses collected on 23 non-consecutive days). Summaries of responses to checklist items are outlined in Additional file 4: Tables S1–S4 (baseline audit, physician and audit responses during intervention).

Compliance with all nine cares improved significantly over time (see Table 3). The largest improvement was for pain management, where the odds of receiving this care during the intervention period compared to baseline (after adjustment for confounders) was 23 times greater, a 42% increase in compliance. Glucose management and head-of-bed elevation also demonstrated much higher compliance rates (increased 22 and 19%, respectively) during the intervention period, with odds ratios (ORs) of 14 and 11, respectively. Medication review also displayed significant improvement with an OR of 10, though the absolute change was only 1.4%.

**Table 3:** Compliance with care processes over time (baseline versus intervention)

	% Absolute change	Baseline (%)	Intervention (%)	Adjusted^a^ odds ratio (95% CI)	P-value
Pain management	42.2	53.4	95.6	22.85 (13.69–38.16)	<0.001
Glucose management	22	75.7	97.7	13.82 (7.01–27.27)	<0.001
Head-of-bed elevation	19	78.3	97.1	10.98 (5.39–22.35)	<0.001
Sedation management	7.5	89.7	97.2	3.89 (1.80–8.42)	0.001
Nutrition assessment	7.4	89	96.4	4.36 (2.4–7.92)	<0.001
Mechanical ventilation weaning	3.6	90.9	94.5	1.92 (1.03–3.59)	0.041
Stress ulcer prophylaxis	3.2	94.4	97.6	3.73 (1.68–8.28)	0.001
DVT prophylaxis	1.7	94.8	96.5	2.24 (1.06–4.70)	0.034
Medication review	1.4	98.4	99.8	9.86 (1.31–74.33)	0.026

‘Not applicable’ and ‘not ventilated’ responses were excluded.

^a^GEE adjusted for patient age, gender, APACHE III severity of illness score, ICU length of stay, vital status upon discharge from ICU, readmission to ICU, type of admission (emergency or elective, post-operative or non-operative, ICU or HDU).

Nutrition assessment (7.4% improvement), sedation management (7.5% improvement) and stress ulcer prophylaxis (3.2% improvement) displayed moderate improvement over time with ORs of 3–5 (see Table 3). DVT prophylaxis and mechanical ventilation weaning demonstrated the least improvement (1.7 and 1.4% respectively) with ORs less than three, although compliance rates at baseline were already very high (95 and 91%, respectively).

At the patient level, of 81 omissions ‘not yet corrected’ during the morning ward round, 64 (79%) were corrected the next day, while 4 (5%) remained as omissions, but were corrected the following day. The remaining 13 cases (16%) were for patients only in the unit for 1 day or on their last day before ICU discharge.

**Fig. 1:**
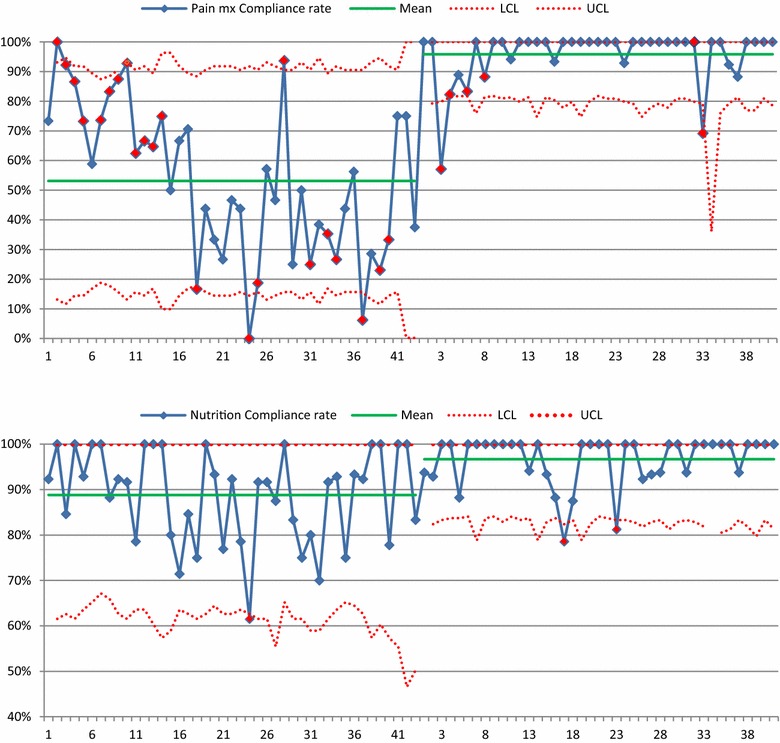
Examples of SPC charts illustrating compliance over time. The first phase is the baseline period, and the second phase the intervention period. *Blue line*—daily unit compliance over time; *green line*—average compliance for each of the two time periods; *red dotted* *lines*—upper and lower confidence (or sigma) limits, i.e. 3 standard deviations either side of the mean; *red diamonds*—SPC rule violations (detailed in Additional file 5: Figures S1–S9).

The SPC charts generated for each care component (examples of charts provided in Fig. 1; see Additional file 5: Figures S1–S9 for all charts with narrative interpretation) illustrated reduced variability in compliance over time for most cares. The only exceptions were DVT prophylaxis and medication management, which as noted above displayed high levels of compliance that was relatively stable over time. Some care components (e.g. pain and sedation management, and weaning off the ventilator) displayed some variability during the first week of the intervention, but then evidence of improvement that was then largely sustained. Despite improvements in compliance, some continued variability for two cares (nutrition and stress ulcer prophylaxis) was noted; both had 2 days where compliance fell below the lower control limit, followed by improved compliance within control limits the next day.

### Checklist concordance with actual delivery of care

The care components with the highest proportion of agreement between physicians and auditors were medications (100%) and stress ulcer prophylaxis (99.57%), while those with the lowest agreement rates were pain (79.23%) and head-of-bed elevation (85.26%). Calculation of both bias indices revealed these data were relatively free of inter-observer bias, although prevalence was high (very high rates of positive responses and very low to zero negative responses) (see Table 4). There were moderate to very high rates of agreement between the two groups, with kappa values ranging from 0.59 for pain management to 0.99 for stress ulcer prophylaxis. Post hoc analyses conducted for each physician designation revealed high levels of agreement (consultant = 0.89, senior registrar = 0.84, registrar = 0.92). Note the smaller number of observations for registrars (n = 316) compared to senior registrars (n = 796) and consultants (n = 930).

**Table 4:** Measures of concordance between physician and auditor checklist responses for each care component

Care component	n	Proportion observed agreement	Bias index	Prevalence index	Byrt’s kappa	Proportion positive	Proportion negative
Medications	289	100	n/a	n/a	No variation^a^	n/a	n/a
Readiness to wean	194	94.33	−0.036	0.933	0.887	0.971	0.154
Glucose management	306	91.18	0.082	0.912	0.824	0.954	0
Nutrition	270	97.04	0	0.956	0.941	0.985	0.333
Stress ulcer prophylaxis	233	99.57	0.004	0.996	0.991	0.998	0
DVT prophylaxis	255	98.82	0.004	0.988	0.976	0.994	0
Head-of-bed elevation	190	85.26	0.126	0.853	0.705	0.920	0
Sedation	150	92.00	0.040	0.92	0.840	0.958	0
Pain	207	79.23	0.130	0.783	0.585	0.883	0.044

Concordance based on 2 × 2 contingency table.

*n/a* not applicable due to no variation in marginal distributions.

^a^Byrt’s kappa not calculated for medications, as there was 100% agreement (no variation between the two respondent groups).

## Discussion

### Key findings

#### Compliance

Compliance with all process-of-care checklist items improved significantly after implementation of the e-checklist, suggesting that its’ use increased medical attention on morning ward rounds, complementing and enhancing routine clinical practices. Cares with the most substantial improvements (pain and glucose management, head-of-bed elevation) had the lowest compliance rates at baseline and the potential for change was therefore greater. In this ICU, the e-checklist had the largest benefit for ensuring maintenance of head-of-bed elevation and managing pain and BSLs within clinically acceptable parameters; given findings of deficiencies in these aspects of care globally, e.g. [25–29], this may be beneficial for many other ICUs.

Reduced daily variations in the care delivered between pre- and post-intervention periods was also evident. Considerable reductions were noted for sedation, weaning from mechanical ventilation and head-of-bed elevation. Improved delivery of these processes for ventilated patients has been associated with decreased ICU length of stay, ventilator days [30] and rates of ventilator-associated pneumonia [31–33]. Use of the e-checklist may reduce practice variations and improve patient outcomes.

With consistently high compliance rates over time, utility of the medications checklist item is questionable. The statement ‘All medications have been checked and reviewed’ appears to have been too broad to provide meaningful data—with only one omission of care during the intervention period. However, with up to 38 adverse events and 498 medication errors noted per 1,000 patient days in ICUs [34], checking and reviewing medications remain an important aspect of ward rounds. In the absence of other improvement strategies, medication reviews should either be integrated into clinical processes as a prompt, or developed as specific checklist items for medications identified as a problem for local units.

#### Concordance

Concordance between clinician and audit responses was high for most care components, indicating that physician responses were reflective of actual care delivery. Three checklist items with kappa values less than 0.85 (pain, sedation, glucose management) contained multi-dimensional statements (e.g. pain required both assessment and management plan/progress review). Physicians may have therefore selected ‘yes’ when one aspect of the checklist item was delivered, suggesting that greater checklist validity could be achieved if each item was unidimensional [35]. This interpretation is, however, not definitive, as contemporary literature has not specifically addressed the issue of multi-dimensional checklist items.

Lower agreement for pain management may have also been due to lack of an agreed, standardised, objective pain assessment, particularly for non-communicative ICU patients, [36] leading to differences in responses (auditors indicated a higher rate of omissions). For audit data, one-fifth of compliant cases were not documented correctly or completely during the intervention, despite pain assessments taking place, an issue previously reported for Australian and New Zealand ICUs [4] and in emergency departments in the USA [37]. It may have been difficult for auditors to assess whether appropriate care was delivered. Similarly, minor discrepancies in concordance for sedation may have also been due to lack of documentation by clinical staff [38–40]. Concordance with blood glucose management may have been impacted by difficulties in maintaining levels within defined limits [41].

Head-of-bed elevation also had low concordance, resulting perhaps from variations in measurement of the angle, e.g. inclinometer measured in 5° increments, differences in the site of measurement of the angle due to the patient’s body position on the bed, and clinicians using personal judgement on angle (overestimates head-of-bed elevation [42]) rather than using the measurement device. Changes in patient position in the bed could also have occurred between the ward round and audit.

### Study strengths and limitations

This study sought to address limitations of previous intervention studies utilising checklists in clinical practice [11]. Methodological strengths included prospective, electronic data collection at the point of care during both baseline and intervention periods. Process measures were based on physician and audit responses using the e-checklist and a multi-faceted approach to daily compliance measurement. A high level of concordance between physician and auditor responses provided evidence in support of the e-checklist’s construct validity. Details on whether an omission of care was corrected upon or after detection were also obtained via the e-checklist. All omissions detected led to care delivery according to subsequent checklist responses. This provides further confirmation that the checklist functioned as intended—to ensure delivery of essential care once omissions are detected.

A before–after study design precluded establishing a causal relationship between e-checklist use and improvement in the delivery of care, although there were factors that supported use of this design: patient cohorts were equivalent, no other unit-level changes contributed to changes in clinical practice at the time, improvement was demonstrated across all care components of the e-checklist and acceptable levels of concordance with audit data were noted. Although the study was carried out over a relatively short period of time, limiting evaluation of sustainability, proof of concept for use of an e-checklist in an ICU setting was established. While this was a single-site study, the sample size exceeded requirements to detect significant differences in compliance over time. An equivalent number of patients pre- and post-intervention with similar demographic and clinical characteristics demonstrated a good representation of the ICU patient population. Study findings could therefore apply to other general combined ICU/HDUs with similar patient demographics.

Inclusion of ‘not applicable’ responses to the e-checklist allowed clinicians to exercise their clinical judgement; not restricting them to a ‘yes’ or ‘no’ response may have compromised accurate measurement and their acceptance of the tool. Patient safety was also an important consideration, with emphasis on delivering care where applicable, ensuring that unnecessary and potentially harmful treatments were not delivered to patients [43]. The ‘not applicable’ response was however not included in compliance measurement, as study outcomes were the delivery of applicable care (and acknowledging that some care items were not always appropriate for individual patients). Measurement of concordance used a 2 × 2 contingency table (two respondent groups and two response options), so it was not possible to determine whether exclusion of ‘NA’ responses impacted on either concordance or compliance measurements.

Also, note that our study aims and procedures precluded any physician responses during baseline measures; therefore, comparisons between physicians and auditors were only available during the intervention phase, while compliance between baseline and intervention phases was assessed by auditor responses. Although concordance levels between physicians and auditors were acceptable (see Table 4), as highlighted in Additional file 4: Table S4, auditors recorded a higher rate of ‘NA’ responses than physicians, particularly for pain, DVT prophylaxis, nutrition and sedation management. The reason for this is not known; possible explanations include differences in checklist completion times and interpretation of ‘NA’ response; physicians might not have realised that ‘NA’ responses were subsumed under the ‘No’ category (i.e. as a reason why care was not delivered); or different interpretations for checklist definitions and instruction for use, despite a data dictionary being available. It is therefore unclear whether these systematic differences in responses influenced our findings.

The Hawthorne effect may have also influenced our findings; although physicians were not provided with project information until after baseline data were collected, there may have been heightened awareness associated with the audits after rounds were completed. As a quality improvement initiative, the intervention provided physicians with useful clinical information and facilitated their engagement with the project. Physicians were therefore aware of the main study aim (i.e. improve compliance with certain cares) and this may have influenced their behaviour during the intervention period: for example, reducing the number of care ‘omissions’ and increasing those classified as ‘not applicable’ for a patient. Although both baseline and intervention periods were treated similarly, it is unknown whether comparable results would be obtained beyond the confines of the study or whether they would be generalisable to other settings.

Finally, with the constantly evolving nature of technology, smartphones and other handheld devices have superseded PDAs since this study was completed.

### Implications for practice or policy

For clinicians, this study demonstrated that use of an electronic checklist that encourages daily assessment of essential cares by senior physicians is associated with improved care delivery. The need for a similar tool or process in other ICUs can be determined by the presence of both patient-level and unit-level variability in the delivery of care, identified by post-ward round audits of practice. The versatility of an e-checklist was also demonstrated, with use: as a clinical support tool; in real-time measurement at the patient bedside; and for auditing care delivery. ICUs can therefore implement e-checklists in different ways depending on their needs, available resources and what practice improvements they wish to achieve.

Continued advances in health-care technologies will impact on the use of e-tools in practice, including clinical information systems (CIS) where checks can be automated with alerts via bedside monitors or messaging services to email accounts or smart phones. Automated content (e.g. intravenous fluids) could therefore be included in a ward round checklist for sign-off by appropriate clinicians, to ensure all relevant aspects of patient care are reviewed. A ward round checklist could be built into a CIS that requires clinician interaction, particularly for aspects of care that cannot be automated (e.g. measuring head-of-bed elevation). It is therefore important to ensure that clinical support tools such as the e-checklist are as robust and flexible as possible; the ability to transfer and adapt them from one platform to another or from one clinical setting to another would broaden its appeal and have the potential to make even greater impact on the quality and consistency of patient care.

Policy makers and service administrators need to consider the process involved in achieving improvements in care delivery and ensure that suitable resources are available. The existence and promulgation of guidelines and policies are insufficient for achieving improvements at the local level [44–46], hence the need for innovative clinical support tools. New QI projects that involve implementing such tools require sufficient time and resources beyond current base ICU funding; conversely, providers also need to be mindful of developing systems and processes that are sustainable without ongoing additional resources.

### Recommendations for further research

Some findings and related issues require further research. Evaluation of the effect that multi-dimensional compared to unidimensional checklist items have on process-of-care measurement would provide clarity around the development of future checklist statements. Given the complex nature of pain and sedation management in ICU and related measurement challenges, further exploration of their essential components and the relationship between the two may assist in evaluating both compliance with these cares and contribute to improving the validity and utility of measures. Further research might also address limitations of this study such as evaluating reliability of the checklist—particularly, inter-rater reliability, the impact of ‘not applicable’ responses and conducting a larger multi-centre study utilising a stepped-wedge trial design [47], ensuring adequate power to detect significant differences in patient outcomes over time. Finally, given continuing advances in technology, further work and study of the different modalities of delivering an e-checklist tool, e.g. incorporation into a unit’s clinical information system (CIS), or as an ‘app’ for tablets or smartphones is also warranted.

## Conclusion

This single-site before–after prospective intervention study demonstrated improved delivery of essential daily care processes after implementation of an e-checklist on the ICU morning ward rounds. Increased compliance and reduced variability in cares delivered over time offered evidence supporting the e-checklist as a tool that may assist in standardising and ensuring the delivery of important elements of patient care.

There was acceptable agreement between physician and independent audit responses, providing evidence of the validity of the e-checklist. In addition to having clinical utility, the e-checklist also functioned effectively as an audit tool. As different ICUs and other clinical settings have unique requirements, there is a need to test different modes of delivering the e-checklist, such as incorporating it into a CIS or using handheld technology to suit the needs of users. While these study findings demonstrate the benefits of using an e-checklist in clinical practice, further work is required to ensure that such tools are robust, sustainable and have clinical utility across a range of practice settings.

## Additional files

Additional file 1: Outline of study procedure; includes activities and purpose of activity by each project stage. Additional file 2: Outline of how key observations were integrated into the study method. Additional file 3: Additional details describing data analyses; includes further description of Generalised Estimating Equations, Statistical Process Control, Byrt’s kappa, Bias and Prevalence Indices, Positive and Negative agreement. Additional file 4:
**Tables S1–S4.** Tables containing detailed checklist responses; includes checklist responses provided by auditors during baseline data collection phase (Table S1), checklist responses provided by physicians during intervention phase (Table S2), checklist responses provided by auditors during intervention phase (Table S3), and comparison of the proportion of checklist responses provided by physicians and auditors during the intervention phase (Table S4). Additional file 5:
**Figures S1–S9. **Statistical process control charts for each care component (Figures S1 to S9); includes graph, rule violations and interpretation.

## Abbreviations

APACHE: Acute Physiology and Chronic Health Evaluation Score
CIS: clinical information system
DVT: deep venous thrombosis
GEE: generalised estimating equations
HDU: high dependency unit
ICU: intensive care unit
OR: odds ratio
PDA: personal digital assistant
QI: quality improvement
SPC: statistical process control
