# Supplementary material for: Testing the implementation of an electronic process-of-care checklist for use during morning medical rounds in a tertiary intensive care unit: a prospective before–after study
Source: Ann Intensive Care. 2015 Aug 4;5:20. doi: 10.1186/s13613-015-0060-1 (PMC4523566; doi:10.1186/s13613-015-0060-1)
Supplement: Additional file 1: — Outline of study procedure; includes activities and purpose of activity by each project stage. [file 13613_2015_60_MOESM1_ESM.docx]

**Additional File 1: Outline of study procedure**

| **Stage** | **Activity** | **Purpose** |
| --- | --- | --- |
| Pre-baseline | 2 senior intensivists invited & consulted to serve as local clinical champions | Serve as drivers of change, assist with clinician buy-in, and have involvement in the planning, development & implementation of the study (e.g. assistance with data definitions) |
|  | Develop data definitions | Consistent use and interpretation of e-checklist items |
|  | Engage research nurses in audit data collection | Provide input into audit procedures, the audit tool, further development of instruction booklets |
|  | One-on-one e-checklist training for all baseline data collectors | Familiarise and provide instruction on use of the e-checklist audit tool (including data definitions) and the checklist server (including live demonstration and practice runs) |
|  | Observations of morning ward rounds | Identify the clinical practices that may impact on the delivery of care to ICU patients during ward rounds; note any considerations that need to be factored into the study intervention/protocol |
|  | Software development and testing | Ensure the e-checklist works as intended; improve performance of the technology |
| Baseline | Audit of the morning ward rounds – process-of-care data collected by research nurses using the e-checklist audit tool | Identify actual practice on a daily basis during ward rounds |
|  | Baseline data extracted & summarised using statistical process control charts | Provide information on delivery of care prior to intervention; generate feedback reports to inform clinicians prior to the intervention stage |
| Pre-intervention | Information sessions to all ICU staff | Present the project in general terms; keep staff informed of changes to ward rounds i.e. use of PDAs & checking mechanism to be introduced at the end of each patient assessment |
|  | Presentation to medical staff | Obtain buy-in from physicians, share results of the baseline audit; obtain consent from participants |
|  | One-on-one e-checklist training for all medical participants | Familiarise and provide instruction on use of the e-checklist tool including live demonstration and practice runs; discussion of checklist statements and related data definitions |
|  | One-on-one e-checklist server training for ward clerks | Updating the patient list in the e-checklist server prior to the morning medical handover and ward rounds |
|  | Provide intensivists, research nurses, & ward clerks instruction booklets (paper & electronic) | Enable ready access to project-related information when required |
|  | Software refinements & final testing of e-checklist software, server & wireless connection | Ensure all components were functional |
| Intervention | E-checklist tool implemented | Use of the e-checklist was the intervention being tested |
|  | Real-time summary reports | Provided clinicians with compliance data displayed using tables and charts via the web portal |
|  | Provide fortnightly feedback reports to clinicians – via email to participants and displayed on notice boards in staff common areas | Quantify the delivery of processes-of-care on ward rounds; generate process data in a format that was easily interpretable, informing clinicians about their practice; enable ICU clinicians to identify areas for improvement |
|  | Post-ward round audits completed by a research nurse four days a week (included one day of the weekend) | Verification of physician responses (validity testing) |
