# Supplementary material for: Testing the implementation of an electronic process-of-care checklist for use during morning medical rounds in a tertiary intensive care unit: a prospective before–after study
Source: Ann Intensive Care. 2015 Aug 4;5:20. doi: 10.1186/s13613-015-0060-1 (PMC4523566; doi:10.1186/s13613-015-0060-1)
Supplement: Additional file 2: — Outline of how key observations were integrated into the study method. [file 13613_2015_60_MOESM2_ESM.pdf]

Additional File 2: Outline of how key observations were integrated into the study method

**Key observations**	**How integrated into study method**
Role of handover versus role of ward round	Use of e-checklist during morning ward rounds confirmed as the most suitable time
After handover medical staff split evenly (in terms of numbers and roles) into two groups – 1 team per pod	Two devices were required to be used concurrently, the e-checklist server was required for collection of data from both devices, and business rules were devised to ensure one source of truth for the collated data
Ward rounds lead by most senior physician	Emphasis placed on engaging senior physicians in the project, training them on e-checklist use, and requesting they lead and/or encourage its use
Roles of the medical team during ward rounds differed depending on clinical lead	All senior medical staff (registrars, senior registrars, consultants) were provided with individual logins for the e-checklist
Ward round flow and content covered	In order to accurately reflect care delivered during ward rounds and to be a useful checking mechanism, use of the e-checklist at the end of the patient visit was deemed most appropriate
Regular disruptions to the ward round	Provided a supporting argument to the need for improvement and possible utility of e-checklist – examples were fed back to clinicians during information sessions

Processes-of-care covered by the e-checklist seemingly not addressed for all patients during ward rounds (despite some apparent attempts to do checks from memory alone)	As above
All processes-of-care were attended to during ward rounds across total observation period	Confirmed content validity work in the clinical setting
Senior medical staff tended to remain at the bedside more often than other staff during ward rounds	Provided additional confirmation that senior medical staff should lead the use of e-checklist
Existing technology in the unit not used routinely e.g. COWs were not necessary to patient assessments, nor as conveniently portable as a handheld device	Provided confirmation there was no existing IT infrastructure suitable for the delivery of the e-checklist
Inconsistent and irregular use of the printed ward round tool	Provided further supporting argument to the need for improvement and possible utility of e-checklist – this was fed back to clinicians during information sessions
No other electronic device was used as a checklist of routine cares to be delivered during ward rounds	Provided confirmation of the suitability of delivering the e-checklist via a PDA

Abbreviations: COWs = computers on wheels; IT = information technology; PDA = Personal Digital Assistant.
