# Supplementary material for: Testing the implementation of an electronic process-of-care checklist for use during morning medical rounds in a tertiary intensive care unit: a prospective before–after study
Source: Ann Intensive Care. 2015 Aug 4;5:20. doi: 10.1186/s13613-015-0060-1 (PMC4523566; doi:10.1186/s13613-015-0060-1)
Supplement: Additional file 3: — Additional details describing data analyses; includes further description of Generalised Estimating Equations, Statistical Process Control, Byrt’s kappa, Bias and Prevalence Indices, Positive and Negative agreement. [file 13613_2015_60_MOESM3_ESM.docx]

**Additional file 3: Additional details describing data analyses**

**Generalised Estimating Equations** (GEE) factored in: 1) the time component of this before-after study; 2) the existence of any correlated (non-independent) data [1] such as the repeated measurement of compliance with cares delivered to individual patients; and 3) the inclusion of confounding variables that could impact on the primary outcome (compliance at intervention). Potential confounders were identified from the literature and included patient age, gender, severity of illness score (APACHE III), ICU length of stay, vital status upon discharge from ICU, readmission to ICU (per separation for current hospital stay only), and type of admission (i.e. emergency or elective, post-operative or non-operative, and admitted as an ICU or HDU patient).

**Statistical Process Control** (SPC) charts rule violations [2]:

- - - A single point falls outside the upper or lower control limits;
    - At least two out of three successive values are more than two standard deviations (SD) from the mean on the same side of the centre line;
    - At least four out of five successive values are more than one SD from the mean on the same side of the centre line;
    - At least eight successive values fall on the same side of the centre line;
    - Six consecutive points trending up or down;
    - 14 consecutive points alternating.

**Byrt’s kappa** [3] corrects for bias in marginal distributions, and is presented with the Bias Index (BI) – a measure of bias between ‘observers’ (physician-entered and audit data) present when the marginal distributions for the raters are unequal (BI = 0 when marginal proportions are equal); and Prevalence Index (PI) – a measure of the differences between the overall proportion of ‘yes’ and ‘no’ assessments (PI = 0 when both responses are equally probable). These measures along with both positive and negative agreement (‘Ppos’ / ‘Pneg’) enable clear demonstration of the nature of any relationship between respondent groups (physician versus auditor) for each checklist item. Byrt’s kappa statistics were calculated using a 2x2 contingency table (i.e. ‘yes care delivered or considered’ and ‘no- care not delivered’) using the concord package (version 1.4-9) in RStudio (version 0.97.168; RStudio Inc. Boston, MA, USA); PI, BI, Ppos and Pneg were calculated in Excel using published formulas [3-4]. Byrt’s method of calculating kappa was used as: 1) Cohen’s kappa is subject to bias in some instances and only suitable for fully-crossed designs with exactly two coders [5-6]; and 2) the potential for checklist data to have a prevalence bias with a larger proportion of observed ratings falling under one category was identified – this type of bias would cause Cohen kappa estimates to be unrepresentatively low [3, 6].

**References**

1. Garson D (2013) Generalized Linear Models & Generalized Estimating Equations. Asheboro, NC: Statistical Associates Publishers.

2. Thor J, Lundberg J, Ask J, Olsson J, Carli C, Harenstam KP, et al (2007) Application of statistical process control in healthcare improvement: systematic review. Qual Saf Health Care 16:387-399

3. Byrt T, Bishop J, Carlin JB (1993) Bias, prevalence and kappa. J Clin Epidemiol 46:423-429

4. Cicchetti DV, Feinstein AR (1990) High agreement but low kappa: II. Resolving the paradoxes. J Clin Epidemiol 43:551-558

5. Kraemer HC, Periyakoil VS, Noda A (2002) Kappa coefficients in medical research. Stat Med 21:2109-2129

6. Hallgren KA (2012) Computing inter-rater reliability for observational data: an overview and tutorial. Tutor Quant Methods Psychol 8:23-34
