# Supplementary material for: Testing the implementation of an electronic process-of-care checklist for use during morning medical rounds in a tertiary intensive care unit: a prospective before–after study
Source: Ann Intensive Care. 2015 Aug 4;5:20. doi: 10.1186/s13613-015-0060-1 (PMC4523566; doi:10.1186/s13613-015-0060-1)
Supplement: Additional file 4: — Tables S1–S4. Tables containing detailed checklist responses; includes checklist responses provided by auditors during baseline data collection phase (Table S1), checklist responses provided by physicians during intervention phase (Table S2), checklist responses provided by auditors during intervention phase (Table S3), and comparison of the proportion of checklist responses provided by physicians and auditors during the intervention phase (Table S4). [file 13613_2015_60_MOESM4_ESM.docx]

**Additional file 4**

**Table S1 Checklist responses provided by auditors during *baseline* data collection phase** (n=635)

| **Care component** | **Yes**  **(care delivered & documented)** | **Yes**  **(care delivered but not documented)** | ***Yes***  ***(total)*** | **Not applicable**^a^ | **No**  **(omission of care)** | **Not Ventilated** ^b^ |
| --- | --- | --- | --- | --- | --- | --- |
| Pain | 311 (49.0) | 28 (4.4) | *339 (53.4)* | - | 296 (46.6) | - |
| DVT prophylaxis | 483 (76.1) | 5 (0.8) | *488 (76.9)* | 120 (18.9) | 27 (4.2) | - |
| Readiness to wean | 254 (40.0) | 7 (1.1) | *261 (41.1)* | - | 26 (4.1) | 348 (54.8) |
| Nutrition | 455 (71.7) | 10 (1.6) | *465 (73.2)* | 112 (17.6) | 58 (9.1) | - |
| Glucose management | 461 (72.6) | 20 (3.1) | *481 (75.7)* | - | 154 (24.3) | - |
| Head of bed elevation | 99 (15.6) | 92 (14.5) | *191 (30.1)* | 40 (6.3) | 53 (8.3) | 351 (55.3) |
| Medications^c^ | 487 (76.7) | 7 (1.1) | *494 (77.8)* | 133 (20.9) | 8 (1.3) | - |
| Sedation management | 149 (23.5) | 8 (1.3) | *157 (24.7)* | 460 (72.4) | 18 (2.8) | - |
| Stress ulcer prophylaxis | 442 (69.6) | - | *442 (69.6)* | 167 (26.3) | 26 (4.1) | - |

Figures in brackets are percentages; missing data excluded. ^a^ Includes clinical contra-indications. ^b^ Auto-fill function in response to first checklist question ‘Is the patient mechanically ventilated?’ Sedation management not included at baseline (‘not applicable’ response was used). ^c^ ‘Not applicable’ response to medications checklist item reflected auditors inability to determine whether a review of all medications was completed.

**Table S2 Checklist responses provided by physicians during *intervention* phase** (n=577)

| **Care component** | **Yes**  **(care delivered)** | **Not applicable**^a^ | **No**  **(omission – not yet corrected)** | **No**  **(omission now corrected)** | ***No***  ***(total)*** | **Not Ventilated** ^b^ |
| --- | --- | --- | --- | --- | --- | --- |
| Pain | 504 (87.3) | 50 (8.7) | 21 (3.6) | 2 (0.3) | *22 (3.8)* | - |
| DVT prophylaxis | 522 (90.5) | 36 (6.2) | 6 (1.0) | 13 (2.3) | *19 (3.3)* | - |
| Readiness to wean | 326 (56.5) | - | 17 (2.9) | 2 (0.3) | *19 (3.3)* | 232 (40.2) |
| Nutrition | 541 (93.8) | 16 (2.8) | 13 (2.3) | 7 (1.2) | *20 (3.5)* | - |
| Glucose management | 562 (97.4) | 2 (0.3) | 4 (0.7) | 9 (1.6) | *13 (2.3)* | - |
| Head of bed elevation | 334 (57.9) | 2 (0.3) | 5 (0.9) | 5 (0.9) | *10 (1.7)* | 231 (40.0) |
| Medications | 576 (99.8) | - | - | 1 (0.2) | *1 (0.2)* | - |
| Sedation management | 313 (54.2) | 23 (4.0) | 9 (1.6) | - | *9 (1.6)* | 232 (40.2) |
| Stress ulcer prophylaxis | 480 (83.2) | 85 (14.8) | 6 (1.0) | 6 (1.0) | *12 (2.1)* | - |

Figures in brackets are percentages; missing data excluded.

^a^ Includes clinical contra-indications.

^b^ Auto-fill function in response to first checklist question ‘Is the patient mechanically ventilated?’ Included ‘sedation management’.

**Table S3 Checklist responses provided by auditors during *intervention phase*** (n=333)

| **Care component** | **Yes**  **(care delivered & documented)** | **Yes**  **(care delivered but not documented)** | ***Yes***  ***(total)*** | **Not applicable**^a^ | **No**  **(omission of care)** | **Not Ventilated** ^b^ |
| --- | --- | --- | --- | --- | --- | --- |
| Pain | 137 (41.4) | 62 (18.7) | *199 (60.1)* | 95 (28.7) | 37 (11.2) | - |
| DVT prophylaxis | 271 (81.6) | - | *271 (81.6)* | 58 (17.5) | 3 (0.9) | - |
| Readiness to wean | 195 (58.7) | 6 (1.8) | *201 (60.5)* | - | 3 (0.9) | 128 (38.6) |
| Nutrition | 258 (77.9) | 21 (6.3) | *279 (84.3)* | 43 (13.0) | 9 (2.7) | - |
| Glucose management | 284 (85.5) | 7 (2.1) | *291 (87.7)* | 8 (2.4) | 33 (9.9) | - |
| Head of bed elevation | 117 (35.1) | 59 (17.7) | *176 (52.9)* | 3 (0.9) | 26 (7.8) | 128 (38.4) |
| Medications | 296 (89.4) | 8 (2.4) | *304 (91.8)* | 27 (8.2) | - | - |
| Sedation management | 144 (43.4) | 11 (3.3) | *155 (46.7)* | 42 (12.7) | 10 (3.0) | 125 (37.7) |
| Stress ulcer prophylaxis | 256 (77.1) | - | *256 (77.1)* | 73 (22.0) | 3 (0.9) | - |

Figures in brackets are percentages; missing data excluded. Audits conducted four days per week.

^a^ Includes clinical contra-indication.

^b^ Auto-fill function in response to first checklist question ‘Is the patient mechanically ventilated?’ Included ‘sedation management’.

**Table S4 Comparison of the proportion of checklist responses provided by physicians and auditors during *intervention phase***

| **Care component** | **Yes** | | **No** | | **Not applicable**^a^ | |
| --- | --- | --- | --- | --- | --- | --- |
|  | **Physician** | **Auditor** | **Physician** | **Auditor** | **Physician** | **Auditor** |
| Pain | 87.3 | 60.1 | 3.8 | 11.2 | 8.7 | 28.7 |
| DVT prophylaxis | 90.5 | 81.6 | 3.3 | 0.9 | 6.2 | 17.5 |
| Readiness to wean | 56.5 | 60.5 | 3.3 | 0.9 | - | - |
| Nutrition | 93.8 | 84.3 | 3.5 | 2.7 | 2.8 | 13.0 |
| Glucose management | 97.4 | 87.7 | 2.3 | 9.9 | 0.3 | 2.4 |
| Head of bed elevation | 57.9 | 52.9 | 1.7 | 7.8 | 0.3 | 0.9 |
| Medications | 99.8 | 91.8 | 0.2 | - | - | 8.2 |
| Sedation management | 54.2 | 46.7 | 1.6 | 3.0 | 4.0 | 12.7 |
| Stress ulcer prophylaxis | 83.2 | 77.1 | 2.1 | 0.9 | 14.8 | 22 |

Figures are percentages; missing data excluded. Checklists completed by physicians daily. Audits conducted four days per week.

^a^ Includes clinical contra-indication
