# Supplementary material for: Testing the implementation of an electronic process-of-care checklist for use during morning medical rounds in a tertiary intensive care unit: a prospective before–after study
Source: Ann Intensive Care. 2015 Aug 4;5:20. doi: 10.1186/s13613-015-0060-1 (PMC4523566; doi:10.1186/s13613-015-0060-1)
Supplement: Additional file 5: — Figures S1–S9. Statistical process control charts for each care component (Figures S1 to S9); includes graph, rule violations and interpretation. [file 13613_2015_60_MOESM5_ESM.docx]

**Additional file 5**

**Figure S1 Compliance with pain management over time**

| **Rule violations** | | |
| --- | --- | --- |
| **Study period** | **Day no.** | **Violations for points** |
| Baseline | 2, 28 | > +3 sigma |
|  | 3-4,  9-10 | 2pts of last 3 above +2 sigma |
|  | 4-5,7-8,  9-10,12 | 4pts of last 5 above +1 sigma |
|  | 8-14,25 | 8 consecutive pts ^ centre line |
|  | 18, 24, 37 | < -3 sigma |
|  | 25, 31, 39 | 2pts of last 3 below -2 sigma |
|  | 33-34, 40 | 4pts of last 5 below -1 sigma |
| Intervention | 3, 33 | < -3 sigma |
|  | 4, 6 | 2pts of last 3 below -2 sigma |
|  | 6, 8 | 4pts of last 5 below -1 sigma |
|  | 32 | 8 consecutive pts ^ centre line |

Interpretation: Daily compliance during baseline measurement was highly variable with average compliance just over 50%. During the intervention period average compliance increased and variability reduced dramatically with few process violations when compared with baseline, and a 69% increase in the number of fully compliant days.

**Figure S2 Compliance with DVT prophylaxis over time**

| **Rule violations** | | |
| --- | --- | --- |
| **Study period** | **Day no.** | **Violations for points** |
| Baseline | 7 | 2pts of last 3 below -2 sigma |
| Intervention | 13, 14 | 8 consecutive pts ^ centre line |
|  | 28 | < -3 sigma |
|  | 28 | 2pts of last 3 below -2 sigma |

Interpretation: High compliance levels at both times that was relatively stable and within control. Slight improvements in daily compliance during the intervention period, as illustrated by the mean line.

**Figure S3 Compliance with nutrition management over time**

| **Rule violations** | | |
| --- | --- | --- |
| **Study period** | **Day no.** | **Violations for points** |
| Baseline | 24 | < -3 sigma |
| Intervention | 17, 23 | < -3 sigma |

Interpretation: Improved levels of compliance, with reduced variability the during intervention period is evident, despite two days where unit compliance fell below 3 sigma limits – each instance was followed by improved compliance (within 3 sigma limits) the following day. There was a 33% increase in fully-compliant days from baseline to intervention.

**Figure S4 Compliance with glucose management over time**

| **Rule violations** | | |
| --- | --- | --- |
| **Study period** | **Day no.** | **Violations for points** |
| Baseline | 12 | 4pts of last 5 above +1 sigma |
|  | 20-21 | 2pts of last 3 below -2 sigma |
|  | 38-39 | 8 consecutive pts below centre line |
| Intervention | 19 | < -3 sigma |
|  | 27-32, 41 | 8 consecutive pts ^ centre line |

Interpretation: Dramatic reduction in variability during intervention period with the majority of days achieving 100% compliance representing a 69% increase in fully-compliant days.

**Figure S5 Compliance with head of bed elevation over time**

| **Rule violations** | | |
| --- | --- | --- |
| **Study period** | **Day no.** | **Violations for points** |
| Baseline | 28 | < -3 sigma |
|  | 28 | 2pts of last 3 below -2 sigma |
|  | 28 | 4pts of last 5 below -1 sigma |
| Intervention | 13 | < -3 sigma |
|  | 14 | 2pts of last 3 below -2 sigma |
|  | 40, 41 | 8 consecutive pts ^ centre line |

Interpretation: Reduced variation in compliance is evident for the intervention period. The two consecutive ‘outlier’ data points (outside -2 to -3 sigma limits) during the intervention period were followed by consistently high levels of compliance. There was a 45% increase in fully-compliant days.

**Figure S6 Compliance with medications management over time**

| **Rule violations** | | |
| --- | --- | --- |
| **Study period** | **Day no.** | **Violations for points** |
| Baseline | 8-10, 20-24 | 8 consecutive pts ^ centre line |
|  | 12 | 2pts of last 3 below -2 sigma |
|  | 33 | < -3 sigma |
| Intervention | 5 | < -3 sigma |
|  | 13-41 | 8 consecutive pts ^ centre line |

Interpretation: Daily compliance during the baseline period was high, although there were several days where compliance dropped off. Only one instance was evident early in the intervention period, with 100% compliance recorded for the remainder of the time.

**Figure S7 Compliance with sedation management over time**

| **Rule violations** | | |
| --- | --- | --- |
| **Study period** | **Day no.** | **Violations for points** |
| Baseline | 33-34 | 8 consecutive pts ^ centre line |
|  | 41 | < -3 sigma |
|  | 41 | 2pts of last 3 below -2 sigma |
| Intervention | 3, 8 | < -3 sigma |
|  | 16-32 | 8 consecutive pts ^ centre line |

Interpretation: Despite high average compliance rates during baseline the need for improvement was evident with two days displaying 50% compliance and one day zero compliance. During the intervention period the majority of days achieved 100% compliance (a 22% increase from baseline i.e. from 64-86%), with a subsequent improvement in average compliance noted.

**Figure S8 Compliance with stress ulcer prophylaxis over time**

| **Rule violations** | | |
| --- | --- | --- |
| **Study period** | **Day no.** | **Violations for points** |
| Baseline | 6, 7 | 4pts of last 5 below -1 sigma |
| Intervention | 16, 37 | < -3 sigma |
|  | 32 | 8 consecutive pts ^ centre line |

Interpretation: Although average daily compliance improved from baseline to intervention, some variability continued during the intervention period. Notably, when compliance fell below the lower control (-3 sigma) limit, it returned to within control limits the following day. There was a 22% increase in fully-compliant days during the intervention period.

**Figure S9 Compliance with management of weaning off mechanical ventilation over time**

| **Rule violations** | | |
| --- | --- | --- |
| **Study period** | **Day no.** | **Violations for points** |
| Baseline | 7 | < -3 sigma |
| Intervention | 2-4 | < -3 sigma |
|  | 3-5 | 2pts of last 3 below -2 sigma |
|  | 5 | 4pts of last 5 below -1 sigma |
|  | 13-25, 36-37 | 8 consecutive pts ^ centre line |

Interpretation: Considerable variability was evident during baseline. After a slow start to the intervention period, compliance improved dramatically displaying consistently high daily compliance rates for the remainder of the study period, including a 24% increase in fully-compliant days.
